# Wildlife Farms, Stigma and Harm

**DOI:** 10.3390/ani10101783

**Published:** 2020-10-01

**Authors:** Jessica Bell Rizzolo

**Affiliations:** Department of Fisheries and Wildlife, Michigan State University, East Lansing, MI 48824, USA; jessica.b.rizzolo@gmail.com

**Keywords:** wildlife farming, conservation, ethics, animal welfare, wildlife trade, stigma

## Abstract

**Simple Summary:**

Wildlife farming is a practice where wild animals are legally bred for consumption in a manner similar to agricultural animals. This is a controversial conservation practice that also has implications for animal welfare and human livelihoods. However, most work on wildlife farming has focused solely on conservation or animal welfare rather than considering all of these ethical factors simultaneously. This paper uses interview data with academics to analyze (a) the harms and benefits of wildlife farms and (b) how wildlife farms are labeled detrimental (stigmatized) or acceptable. Results indicate that consideration of the harms and benefits of wildlife farms incorporate conservation, animal welfare, scale disparities between sustenance and commercial farms, consumer preferences, species differences, the dynamics of demand for wildlife products, and governance. Whether wildlife farms are stigmatized or accepted is influenced by different social constructions of the term “wildlife farm”, if there is a stigma around use of a species, a form of production, or the perceived quality of a wildlife product, cultural differences, consumer preferences, geopolitical factors, and demand reduction efforts. This paper discusses how the ethics of wildlife farms are constructed and how shifts in context can alter the ethical repercussions of wildlife farms.

**Abstract:**

Wildlife farming, the commercial breeding and legal sale of non-domesticated species, is an increasingly prevalent, persistently controversial, and understudied conservation practice. The adoption or rejection of wildlife farms is a complex process that incorporates numerous ethical considerations: conservation, livelihoods, animal welfare, and cultural practices. This paper uses qualitative interview data with key informants (academics) to analyze (a) the harms and benefits of wildlife farms and (b) the factors that influence whether wildlife farms are stigmatized or accepted. In evaluations of wildlife farming’s harms and benefits, respondents incorporated multiple considerations: animal welfare, environmental impacts, scale disparities between sustenance and commercial farms, consumer preferences, species differences, the substitutability and accessibility of wildlife products, and governance. The results further indicated that the stigmatization or acceptance of wildlife farms is affected by the “wildlife farm” label, if there is a stigma around use of a species, a form of production, or the perceived quality of a wildlife product, cultural differences in wildlife use, wildlife consumer typology, geopolitical factors, and demand reduction efforts. This paper analyzes the complexities of wildlife farming such that stakeholders can understand the impacts of this practice on species, human communities, individual animals, and the legal and illegal wildlife trades.

## 1. Introduction

Wildlife farming is a broad term that encompasses a variety of contexts and species [1]. However, wildlife farms often include three aspects: (a) commercial intent (the production of wildlife for sale), (b) the breeding of a species, and (c) the use of a non-typical wild species that is beyond the traditional domestic animal [2]. The species used in wildlife farming involve fauna from every taxon [1] as well as flora [3]. Birds are farmed for the pet trade, reptiles (e.g., turtles and snakes) for both food and pets, frogs and shrimp for food, porcupines for meat, crocodiles for their skin, bears for their bile, and tigers for their bones (both used in Traditional Chinese Medicine). While most of these animals are killed prior to becoming commodities, some (often birds and reptiles) are traded live for the pet trade.

While the terms “wildlife farming” and “wildlife ranching” are sometimes used simultaneously, these production systems tend to be different. “Wildlife farming typically refers to intensive management and husbandry of wild stock, and wildlife ranching usually refers to less-intensive management in semi-free ranching contexts” [4] (p. 1224). Wildlife ranching is common on the African continent, but less so in China and Southeast Asia, where wildlife farming is widespread [5,6,7]. While production systems vary, “wildlife farming” is usually assigned that term due to its replication of the techniques used in intensive agriculture, such as a high density of animals in a small, closely managed space. Many of the arguments both for and against wildlife farming focus on this element of intensive confinement; for example, these systems can use land more productively than other wildlife extraction methods [8] but often have severe animal welfare consequences [9]. 

Since wildlife farms involve the legal production, trade, and/or consumption of live wildlife or wildlife products, they are a form of sustainable use and are subject to larger deliberations over that approach versus a more protectionist orientation [10,11]. These debates are complex but generally focus on conservation, livelihoods, or animal welfare. From a conservation perspective, wildlife farms are considered a form of supply-side conservation, which refers to practices meant to provide wildlife products in a more sustainable way [3]. For many wildlife species, current rates of consumption are unsustainable [12] and regulatory and enforcement actions insufficient [13]. Some conservationists have proposed wildlife farming as a potential tool for saturating the demand for wildlife products and reducing poaching. However, in order to improve species’ conservation status, wildlife farming needs to meet various conditions that involve both biological characteristics of the species and the mechanisms of demand [3]. Major requirements include, but are not limited to, the farmed product functioning as a replacement for the wild product (substitutability), a lack of laundering (which may occur when wild-caught animals are falsely labeled as captive-bred), no restocking of the farmed stock with wild animals, farming being more cost-efficient than poaching, and demand remaining constant [1]. 

It is rare for these criteria to be met [1,14]. Often, dual markets develop for wild-caught and farmed products [3], as consumers tend to prefer and be willing to pay more for wild-caught wildlife [15,16,17]. However, the wild/farmed nexus is complex and stated preferences may or may not align with actual market dynamics [18]. Legal wildlife farms can also launder animals for the illegal wildlife trade [19]. Particularly for species that do not breed well in captivity, such as bears, wildlife farms are often filled either partially or fully with wild-caught animals [20]. Although risk perceptions of poaching are complex [21], there is evidence to suggest that, particularly for high-value species such as tigers, the risks of poaching continue to be low relative to the rewards/profits and this can undermine legal trade [17]. In addition to this economic-based argument, other aspects of conservation are also debated within the context of wildlife farming. The release of farm-bred wildlife into the wild is a contentious issue, with some citing it as a potential benefit of wildlife farms [8], while others contend that, at least for some farmed species, this is a dubious conservation method [22]. In areas of the world where reliance on wild meat or bushmeat for protein is high, wildlife farms have been evaluated as a potential alternative to sustenance hunting [23] and as a method for shifting consumption patterns to more harvest-tolerant species [24].

While much of the scientific debate over wildlife farms has occurred in the conservation literature, other elements are also relevant to the discussion. Wildlife farms can provide livelihoods and/or food security to impoverished communities [24,25]. The economic impacts on communities depends upon the species and the geographic context. This impacts whether wildlife farms are viewed as more or less efficient than hunting or the production of domestic species/crops [23,26]. Finally, another cluster of arguments around the use of wildlife farms focuses on animal welfare, particularly of charismatic megafauna such as bears, tigers, and sea turtles [9,22,27]. For example, bears farmed for bile are kept alive for many years, often in cages where they can only move their head, with an open wound through which bile is procured, which causes severe pain [9]. Unlike wildlife ranches, where wildlife sometimes have freedom of movement, many wildlife farms restrict animals’ space and their expression of natural behaviors, particularly for large carnivores and migratory animals [22,28]. This can result in severe stress and trauma that are often seen as endemic to the commercial production systems in which these animals are confined [22,29].

This paper uses a harm-based approach for its analysis of wildlife farms. A harm-based perspective allows for the consideration of both legal and illegal harms. This is important for two reasons. First, human treatment of wildlife exhibits extreme cross-cultural variability—a legal practice in one country might be classified as animal abuse in another. A focus on harm casts a broad net that can encompass diverse practices. Second, the notion of harm includes effects of wildlife farms on individual animals, or what is known in critical criminology as species justice [30,31]. Animal well-being/welfare within the wildlife trade is drastically understudied and requires more integration with the conservation literature [32]. While wildlife farms can have severe and detrimental impacts on individual animals [33], these harms are often legal. A harm-based approach allows for a critical examination of how certain harms against wildlife are stigmatized while other harms are deemed acceptable [34]. It should be acknowledged that, while this approach is intended to counteract the ways in which numerous harms against wildlife are ignored or neutralized [34], there is an inherent positionality in employing a harm-based perspective, as it presupposes the existence of at least some harms (or negative impacts) of wildlife farms. Further, it is important to note that benefits, like harms, can be either legal or illegal. Both are normative (rather than value-neutral) concepts—whether an effect of wildlife farms is conceived as a “benefit” or a “harm” depends upon the perspective of the observer.

In addition to examining wildlife farming’s perceived harms and benefits, this paper looks at what factors influence the stigmatization or acceptance of wildlife farms. Stigmatization is the process by which an attribute, behavior, or product is marked as “contrary to a norm of a social unit” [35] (p. 80). The notion of stigma is useful for understanding cultural variability in the acceptability, legal status, and perceived impacts of wildlife farming. The presence or absence of stigma around different forms of wildlife consumption is influenced by both the social construction of the commodity (e.g., which parts of wildlife are seen as viable products for consumption) and the social construction of deviance (e.g., which wildlife consumption behaviors are viewed as socially unacceptable). Even the economic value of wildlife products is inextricably linked to social valuation and perceptions. For example, cultural beliefs about the superiority of wild over captive-bred products can exert a heavy influence on the economics of the wildlife trade [36]. This paper looks at how the process of stigmatization affects wildlife farms in diverse cross-cultural contexts.

There are three main gaps in the literature on wildlife farms that this paper addresses. First, it examines how academics frame the issues that surround wildlife farms. Prior work on wildlife farms has looked at wildlife farmers or the public but not at academic researchers themselves. However, scientists and other academics have the power to shape the contours of what is acceptable in wildlife policies [37]. Although there have been well-documented instances of ostensibly scientifically driven policy decisions that were actually made for non-scientific reasons [38], the debate around wildlife farms is still shaped by how researchers set research priorities and evaluate evidence. Therefore, their perspective provides valuable insight into how the meanings of “harm” and “benefit” are constructed. Second, this paper uses a qualitative approach to the analysis of wildlife farms. There is a paucity of qualitative research on wildlife farms in the scientific literature but, given the cultural, legal, economic, and environmental complexities of this topic, a qualitative approach is useful for understanding the dynamics of wildlife farming and its impact on species, communities, and individual animals [39,40].

Finally, while there are existent frameworks for evaluating the conservation claims of wildlife farming [1,3] as well as examinations of the animal welfare implications of wildlife farms [29], the environmental and animal welfare impacts of wildlife farms tend to be examined separately rather than in tandem. This paper analyzes the acceptance or non-acceptance of wildlife farms as a complex process that simultaneously integrates numerous factors: conservation, livelihoods, animal welfare, and cultural practices. Adding to this complexity, acceptance or non-acceptance of wildlife farms can occur at two levels: formal (whether or not governmental institutions legalize wildlife farms) and informal (whether or not societal norms and practices connote acceptance of products from farmed wildlife). Ethics refer to normative considerations of how we *should* act [41]. The overall aim of this paper is to use qualitative interview data with key informants to categorize the different factors that inform the wildlife farm debate. Such information is an essential first step in analyzing the ethical arguments made both for and against wildlife farms [41].

## 2. Methods

This paper used in-depth, semi-structured key informant interviews with eight academics to examine the research questions. Qualitative methods were chosen due to the complexity and novelty of the topic of wildlife farming as well as the importance of respondent-driven narratives in examining the research questions. While in a number of global regions, such as China and South Africa, there have been increases in farmed wildlife due at least in part to government policies [2,42,43], research into wildlife farms is still limited and an emerging area of analysis. Qualitative methods are particularly important in the exploratory phase of research [39]. Qualitative methods focus on depth over breadth, are well-suited to the examination of complex processes, and can elicit insider information that could easily be missed by a pre-set quantitative research instrument [39,40]. 

A semi-structured interview format provides a general framework but also presents an opportunity for the respondents to generate new areas of inquiry, which is particularly important for generating a holistic understanding of an understudied topic such as wildlife farming [39,40]. This research method also places an emphasis on the academics’ own discourse, or *how*, as well as what, content is expressed. Questions are intentionally open-ended so as to provide space for the respondents’ individual interpretations of the research topic. This allows respondents to express uncertainty, complexity, and ambiguity [39,40]. This is important given the intersection of the complex environmental, cultural, legal, and animal welfare aspects of wildlife farms.

The academics in the sample functioned as key informants [44]. Key informants are experts with access to specialized knowledge about a community and/or topic [44]. It is important to note that publication of a peer-reviewed article on a topic does not necessarily connote “expertise”, especially for a novel, evolving, and conceptually vague topic such as wildlife farms. Key informants are identified based on their knowledge of the topic of interest. While this knowledge might be narrowly specialized (e.g., the academic might not know what she/he does not know), collectively, interviews with these respondents are useful for gathering rich, detailed information about new areas of inquiry. Key informants are able to provide complex answers to open-ended questions and are an important starting point for understanding the terrain of a new social issue [45].

The interview questions were divided into three parts (Appendix A), each with a specific aim. The aim of the first section was to explore each respondent’s orientation towards the evaluation of wildlife farms. Each academic was asked to define terms central to the research, such as “conservation”, “wildlife farming”, and “correct treatment of wildlife”. Respondents were also asked which factors they used to evaluate the harms and benefits of wildlife farms in order to see which considerations (e.g., conservation, livelihoods, animal welfare) they include in this decision-making process. The aim of the second section was to evaluate the potential harms of wildlife farms. Respondents were first asked the question, “Does wildlife farming harm wildlife?” (with harm intentionally left undefined in order to elicit their initial thoughts). Follow-up questions asked about potential animal welfare harms in order to address the species justice aspect of wildlife farms [30,31]. The aim of the final section was to understand the complex contextual variables (such as species, cultural attitudes, legal contexts, or environmental impacts) that influence the stigmatization or acceptance of wildlife farming. Respondents were asked about which factors contribute to/maintain wildlife farming, who has the power to decide if wildlife farming is made legal or illegal, how cross-cultural interaction affects the acceptance or stigmatization of wildlife farms, and to describe any stigma(s) that exist around wildlife farms. All questions were open-ended and were intended to initiate rather than direct the course of the interview.

The sample was generated through Google Scholar, a leading platform for scientific discourse and exchange of scientific articles (the accuracy of the resultant data was verified through the search system of the Michigan State University Library). This was used to generate a list of academics who had published on wildlife farms in peer-reviewed scientific journals. The researcher searched for articles published in English from 2010 to 2019 under the search terms “wildlife farming”, “bear farming”, “tiger farming”, “snake farming”, and “turtle farming”. The use of species-specific search terms was to ensure that the broad term “wildlife farming” was not biased towards articles on mammals. However, the species-specific terms produced few results that met inclusion criteria. All of the authors in the final sample were found through the more general “wildlife farming” search term. The focus on the term “wildlife farming”, and the exclusion of the terms “game ranching” and “wildlife ranging”, in the sampling frame does introduce an element of geographical bias. Wildlife farming tends to be more common in Asia whereas game or wildlife ranching is more prevalent in North American and South African contexts. However, this paper focuses on “wildlife farming” in order to capture the perceived harms and benefits of the intensive production systems that tend to define these ventures.

Search results that had not undergone peer-review (such as dissertations, news articles, book chapters, and reports from non-governmental organizations) were excluded from analysis. For all remaining results, from the first twenty pages of search results, the first author (or corresponding author, if different) was contacted through an emailed recruitment letter and (for non-responders) a follow-up reminder. Thirty-three academics were contacted and eight agreed to participate, for a response rate of 24%. The term “academic” is used thus forth to refer to the respondents, all of whom publish their work in peer-reviewed academic journals. Although no quotas were set or enforced for gender, the sample that emerged was naturally balanced in terms of gender (50% male and 50% female). The study was deemed exempt by Michigan State University’s Institutional Review Board (study number 00002854).

Although one limitation was a small sample size (of 8 respondents), the variability of the sampling frame was replicated in the sample. The researcher read all of the abstracts in the sampling frame to identify sources of variation and coded for relevant attributes. The abstracts differed in species of wildlife studied, academic discipline of the author, geographic focus, and orientation towards wildlife farming (some abstracts emphasized the harms of this practice while others focused on the benefits). The sampling frame, while small, replicated the variability of these relevant attributes. The respondents had knowledge of a variety of farmed wildlife species from different geographic and ecological milieus (Table 1).

The purpose of these key informant interviews was to generate in-depth narratives from different academic voices that could yield preliminary information about the impacts of wildlife farms and thus inform further research on this topic. The rigor of qualitative research can be evaluated by its dependability, credibility, confirmability, and transferability [46]. The author put in place the recommended safeguards to achieve these attributes, such as reflecting on her own potential biases, tailoring the questions to the experiences of the respondents, and seeking clarification from respondents regarding her interpretations of their words [46].

Due to the geographic range of the participants (who were located in four continents: North America, Europe, Africa, and Asia), interviews were conducted through Skype audio. Skype allows interviews to be recorded and downloaded in a secure format. Skype audio was chosen over video in order to prevent bandwidth issues (some of the participants were in remote locations with a poor Internet connection) and to ensure consistency in how the participants interacted with the interviewer. Due to time differences of up to 12 h between the interviewee and the interviewer, the interviewer sometimes conducted interviews from work and sometimes from home, and the audio format removed the potential influence of interviewer location. While audio/phone interviews can increase social desirability bias, especially in response to sensitive questions, and can alter self-reported behavior, they also allow for a greater geographic reach [47,48]. Audio interviews were deemed appropriate for this research since (a) participants were asked about their research, not about personal behavior, and (b) a wide geographic range was essential to the aims of this paper. All interviews were conducted, recorded (with permission from the respondents), and transcribed by the author for accuracy. Interviews were an average of 45 min (with a range of 30 to 60 min). Due to the relatively small number of interviews, the analysis was done by hand without the aid of software. The author read the transcripts multiple times, developed codes based on reappearing themes, then re-read each respondent’s transcript to identify quotations representative of each code. Codes that could not be supported by quotations were eliminated, and codes with a high degree of overlap were consolidated into broader themes.

## 3. Results

### 3.1. Sample

Respondents were diverse in their academic foci. Their work examined a wide range of species that encompassed both mammals and non-mammals in varied marine and terrestrial environments (Table 1). These species were farmed (or being considered for farming) for different reasons; while some were used for food, others were farmed for Traditional Chinese Medicine. Congruent with high rates of wildlife farming across China and Southeast Asia [6,7], the majority of respondents had expertise in this geographic reason. However, several respondents worked in other geographic contexts such as Africa and Oceania. The results indicate that academic work on wildlife farming intersects with numerous disciplines other than conservation. The respondents self-identified as specialists in a variety of fields. These included conservation-focused disciplines (such as biology, ecology, and conservation social science) as well as fields such as economics, wildlife domestication, and consumer demand. There were no significant differences in responses/themes based upon the gender of the respondent.

While all respondents defined conservation as a process that focused on species-level or population concerns (rather than individual animals), they emphasized different parts of this process (Table 2). Further, when asked how to determine the correct treatment of wildlife, or how to structure human–wildlife interaction, respondents mentioned a variety of factors other than conservation. These considerations included moral, animal welfare, cultural, economic, and criminal factors (Table 3). All of the interviewees mentioned at least two of these factors and discussed their efforts to simultaneously balance numerous, sometimes conflictual, priorities. As views of conservation and the correct treatment of wildlife inform how “harms” and “benefits” in wildlife farming are defined, the respondents addressed this question from various angles. The small sample nevertheless allowed for a rich examination of how different cultural contexts, approaches to conservation, and species differences impact the evaluation of wildlife farming.

Most respondents defined wildlife farming as the commercial breeding of a wild, non-domesticated species. However, as discussed below, the interviewees gave examples of how the label “wildlife farm” is sometimes misleadingly applied or withheld in order to gain social approval for the practices occurring at the facility. Some respondents noted that, while wildlife farming is often perceived as applying only to fauna (animals), the farming of flora (plants) is technically included under the term “wildlife”.

Respondents mentioned that it is important to differentiate wildlife farming from other forms of captive breeding. According to one academic, “there are two kinds of captive breeding: conservation breeding and commercial breeding (or wildlife farming)”. He noted that the commercial aspect of wildlife farming distinguishes it from other forms of captive breeding that have a more explicit conservation purpose, such as breeding animals for release into the wild or for the intent of genetic diversity. Another participant clarified that it is also important to distinguish between “open- and closed-circuit systems” of captive breeding. This refers to, among other aspects, “where the primary stock comes from, and if it is continually enhanced with wild individuals or not”.

Four respondents perceived wildlife farming as expanding overall. However, all the interviewees discussed how the prevalence of this practice depends upon species and country and, as the farming of some species wanes, it emerges for others. One respondent noted that “certain animals will decrease in how much they’re farmed (such as bears in some countries) but now you see a lot of porcupine farms”. Another academic mentioned that, due to social pressure and the ban on international trade in the species, there is only one sea turtle farm remaining (in the Cayman Islands). Some respondents mentioned that, while wildlife farming is increasing in Asia, they expect it to lessen in other geographic contexts over time. One respondent clarified that the increase in farmed wildlife includes species inaccurately labeled as captive-bred, he said that “the trade in species declared as captive-bred is expanding but in parallel with that is laundering of wild-caught species being declared [as] captive-bred, that is also expanding”.

### 3.2. Harms and Benefits of Wildlife Farms

#### 3.2.1. Animal Welfare

The academics used various factors to evaluate the harms and benefits of wildlife farms (Table 4). All of the respondents mentioned the detrimental consequences of wildlife farms on animal welfare. They specifically mentioned signs of extreme stress, food deprivation/low-quality nutrition, dehydration, limited space sometimes to the point where the animal was unable to move, inability to engage in natural behaviors (such as migration or socialization), and the separation of mothers from their young. The academics were not veterinarians but, as first-hand observers of wildlife farms who often worked in interdisciplinary teams with veterinarians, they all brought up animal welfare as a concern. However, as conceptions of animal welfare are wide and variable, justification by a veterinarian is not necessarily required. One respondent who studied bear farms described bears in “tiny rusty cages” with a “catheter inserted in its gallbladder to remove bile, which absolutely shortens their life and I can imagine is painful and uncomfortable and gives them a plethora of diseases”.

Respondents who studied reptiles, as well as those who looked at mammals, brought up animal welfare as a concern. An academic who looked at sea turtle farms explained “the animals were biting each other all the time, [and] they were quite stressed, [and] they weren’t really able to show their natural behaviors. Those are all ways that you can say that animal welfare is being harmed. I’m sure there’s different levels to that… stress levels are probably a good indicator and they were physically attacking each other. There are people who say: ‘well you also see that when you visit a cow farm’ and that might be true, but that doesn’t mean it’s OK from an animal welfare perspective”. Another academic who studied snake farms mentioned welfare abuse that occurs because of the physical resilience of reptiles. Although these animals can survive rough handling and long periods without food or water, “they are suffering as much [as mammals]”.

Several respondents mentioned the difficulty of balancing animal welfare with other concerns (such as livelihoods). As one academic noted: “I don’t think anyone who would say we are going to produce wildlife of any sort would say that we can create enclosures that match wild conditions and some people might say that that’s the only condition under which they would accept wildlife farming. I appreciate that the economics of it, and space and so on, make that fundamentally impossible. Like with most things, I suspect there’s a compromise”. However, some of the academics (particularly those who focused on mammals), noted that the “scale of suffering” reached a point where animal welfare became a paramount factor. One academic who visited bear farms said, “I can’t remove myself from evaluating it in the context of the welfare of the animals, which supersedes anything…for me the welfare dominates everything…I think people who can debate farming, particularly of these large carnivores, they don’t see these terrible farms and they actively remove themselves from having to confront that”.

#### 3.2.2. Environmental Impacts

Interviewees described both the environmental harms and benefits of wildlife farms. Five of the respondents mentioned how it is difficult for wildlife farms to reduce poaching. One respondent noted that, “wildlife farming involves a lot of costs: infrastructure, operating costs, regulatory and certification schemes, all involve a lot of costs, and on the contrary, poaching involves low costs”. Another discussed how, even if wildlife farms lower the cost of wildlife products, this must be combined with higher risks for poachers or this will have counterproductive effects. Of crocodile farms he said: “crocodiles breed well in captivity… that’s not the issue and it may have impacts on the price. But if there’s no risk to the poacher, maybe the price has halved, but all that means is that the poacher now needs to get twice as many animals to continue his income. It doesn’t matter how low the prices go [for farmed products], if there’s no risk, profit is still profit”. A common comment was that “poaching continues as farming continues”. However, the respondents also mentioned that the relationship between wildlife farms and poaching is difficult to prove one way or another because of the complexities of the wildlife trade. As one academic replied: “That’s one of the arguments that I hate: that farming will decrease poaching. It’s used for example in China because there are legal farms in China and the bear populations in China are apparently, we don’t really know for sure, relatively stable; however, that does not take into account all the Chinese poachers coming into Southeast Asia or the tours (illegal wildlife tours going into Southeast Asia) or the international trade coming across”.

The academics also mentioned other environmental considerations such as laundering, waste, genetic pollution, land management, and climate change. One interviewee noted that wildlife farms can open up a “channel for the laundering of illegally sourced animals into the international market; for example, geckos are taken from the wild in Indonesia and laundered to the global market and declared as captive bred and they’re not”. Another noted that the waste of wildlife farms can harm the local ecosystem and gave the example of frog farms in Thailand, which produce “massive amounts of waste because it’s cheaper to produce them [frogs] en masse in very small enclosures”. However, another respondent reported that snakes produce very little waste so “in China, snake farming is the only livestock activity that can be practiced on the edge of rivers because there’s no waste”. One academic mentioned that genetic pollution can occur when farmed animals are released into the wild, either intentionally or accidentally. He noted that farmed wildlife are often “hybridized taxa” that are problematic from a conservation standpoint. Another interviewee discussed the benefits of snake farms from the perspective of land management in a changing climate. He noted that snake farmers promote “pesticide-free rodent management since they want non-poisoned rats to feed their snakes” and discussed how snakes can be raised in a relatively small area, require very little food inputs (snakes are “90% more efficient than warm-blooded animals like chickens or pigs”), and are tolerant of extreme weather events such as drought. Overall, the respondents emphasized how the perceived environmental harms and benefits of wildlife farms vary greatly depending upon the type of production and the species.

#### 3.2.3. Sustenance, Commercialism, and Scale

Two of the respondents studied small-scale wildlife farms geared at the provision of sustenance. One interviewee who studied the domestication of the giant cane rate in Cameroon noted that “the advantage of this animal is that, since it is small, it can be reared by farmers who are poor, who do not have a lot of capital to invest in stables and fences, or whatever you normally need to keep animals”. Similarly, another academic who researched snake farms emphasized how this species is uniquely suitable for small-scale farmers with limited resources, since snakes can be raised on a vertical plane and require minimal and sporadic food. Respondents discussed how sustenance impacted how they weighed the differential impacts of wildlife farms. As one interviewee said: “if you deal with poor farm families who endure every year, over parts of the year, devastating hunger, the question of whether to domesticate an animal or not is a question of whose welfare is more important, the person’s welfare or the animal’s welfare, and then if you can’t satisfy both, I would say it’s the human being that should be given advantage”.

Other interviewees, particularly those with expertise in the farming of bears and tigers for luxury products, viewed wildlife farms as primarily a profit-driven (rather than a conservation-oriented or sustenance-based) enterprise. One noted that “[wildlife farming has] always been for maximum profit, it’s never been for a conservation reason, as least for these large carnivores”. Another said: “there are very few examples where farming has been used primarily as a conservation strategy versus as a commercial thing that may or may not have ancillary conservation benefits…the examples that I can think of that are actually conservation-driven are sea turtles in the Caimans and it’s been tried for orchids”. Another respondent with expertise on sea turtle farms described how these facilities too became commercialized: “when this business was created in the 1960s, the initial rationale behind it was let’s try wildlife farming as a conservation tool… but as they kept doing it, you need to find money to run these things, and at the same time you realize the potential in terms of attracting people to go and visit [so now] it’s more like a theme park along with a wildlife farming facility…it just developed that way because they need money”.

Some respondents also mentioned how the profit motive of wildlife farms (as commercial enterprises) influence other factors such as animal welfare. One interviewee noted that, since sea turtles “take so long to grow and so long to be ready for slaughter, you have to have a lot in order to meet demand. You have to have a lot [of turtles] at the same time because you have different cohorts. And also of course these facilities are restrained by size”. This leads to extreme crowding and stress for the turtles. Another academic mentioned that the profit motive can lead to an expansion of demand, saying that, “when it’s profitable, people will begin using these products in different forms or ways. So let’s say tiger bone was once only used for back pain or whatever it was and now you have tiger wine which is supposed to cure cancer too… that’s how the economy works, if there is a demand and it’s readily available, people will of course try to make more money out of it”.

#### 3.2.4. Demand for Wildlife Products

A primary way through which the academics conceptualized the harms and benefits of wildlife farming was the impact of this practice on demand for wildlife products. Most of the respondents mentioned surging demand for wildlife amidst an extinction crisis as a primary driver of wildlife farms. As one interviewee noted, “if you’re going to supply demand purely by going out and shooting a bear, it’s not going to work, because there’s no bears left. Keeping them in captivity means you have a constant supply of bile”. Some respondents mentioned the saturation of demand as a benefit of wildlife farms (specifically of snake farms).

Other interviewees noted that wildlife farms can increase demand. One academic who researched bear farms noted, “demand is so rampant. These animals are gone so the only way to continue filling this demand is to have them in farms. So, what that means is that you’re fueling this demand, you’re continuing this demand…when the [bear] farming started [in Vietnam], it became this huge fad so it absolutely increased demand. And that demand has maintained. I don’t think it ever decreases demand”. Another academic agreed: “farming often increases demand… For example, the Asiatic black bears in Korea… once the government legalized farming, the bears went extinct in Korea because of that”. One respondent mentioned tigers as another example where farms have failed to decrease demand: “tigers breed like flies in captivity and are still getting completely decimated in the wild in all of their range states so clearly the tiger farms in China haven’t removed any of the demand for wild tiger”.

However, it was common for the respondents to clarify that demand is nuanced and it is difficult to establish direct causation between wildlife farms and demand. As one academic noted: “we don’t know [the impact of wildlife farms on demand for wildlife products] because demand is so variable. Wildlife farming, I think it can decrease demand for wild products if people are happy with substitution. However, I think it does normalize consumption of certain species, which increases demand. But I think it can probably increase demand for the farmed one rather than the wild one…but we also know, and again it depends on the product, if things are rare, there’s going to also be increased demand for them in certain communities”. In addition to considering demand more broadly, the respondents examined accessibility and consumer preferences, species differences, substitutability, and governance.

#### 3.2.5. Accessibility and Preferences

The respondents discussed accessibility as one mechanism through which wildlife farms influence demand. As one academic noted, “wildlife farming could also decrease prices and then it would become available to a larger group of people. With high prices, most people simply couldn’t afford these luxury products”. Another concurred: “now there’s a whole new market available, so demand has gone up”. One interviewee discussed how, when endangered animals are farmed, the volume of products increases, and so can demand: “some of these animal products were very rare in the past and now that they’re being bred on a large scale, more people have access to them, and that already could increase demand”. Another respondent noted that wildlife farms also make procuring wildlife products easier, saying that, at least for wild animals, “it’s been a challenge for someone to poach them… when you remove the farms you remove the easy accessibility and you add in all those challenges”.

Some of the interviewees looked at the impact of wildlife farms on demand in terms of preferences. This included analyzing “if having something [legal and farmed] available was changing people’s attitudes towards the wild or farmed turtle meat or if they were still interested in buying illegal meat”. Another academic noted that, “I think the perception that consuming or purchasing from a farm is not detrimental to wild populations is probably the biggest threat posed by the captive breeding industry”.

#### 3.2.6. Species Differences

All of the interviewees mentioned how the effects of wildlife farms on demand are species-specific. One respondent gave the example of snakes and noted that, for this species, “[farming has] saturated demand… supply outstrips demand massively in the snake trade”. However, he clarified that snakes are resilient to “high levels of harvest”. Respondents who studied other species, such as turtles, that take years to breed and mature, noted how biological factors of the animals restrained the effectiveness of wildlife farms. One interviewee discussed the effects of species differences in depth: “For crocodiles, farming has been legalized for many years and it has relieved the poaching crisis in some countries, not all countries. For crocodiles, the demand is just focused on meat and skin, so the demand has not increased, so I think the crocodile farms have helped relieve the poaching crisis in some countries, but I think for other species, it is a quite different. Bear farming has been legalized for quite a long time, but bear farming has not contributed to reducing poaching of bears from the wild. For bears, there is one market for legal products and one market for illegal products. In Thailand, Laos, and Vietnam, there is a preference for products from wild bears. So, the promotion of bear farming hasn’t reduced the demand for products from wild bears. Farming bears is not a solution to the poaching crisis, and has negatively affected the welfare of bears”. Some respondents discussed how wildlife farms can produce differential results due to biological characteristics of the species, such as time to maturity, ability to breed in captivity, and resilience to harvest. Other interviewees, as evidenced in the contrast drawn between crocodiles and bears, emphasized how it is substitutability (or lack thereof) that produces variable outcomes.

#### 3.2.7. Substitutability

Respondents noted how the impact of wildlife farms depends upon whether farms can provide an alternative (or substitute) for poached wildlife; as one interviewee noted, “if they [consumers] prefer horn from farmed rhinos, then farming rhinos can be a solution, but if more of them prefer horn from wild rhinos, then farming rhinos might not be a solution”. However, five interviewees mentioned consumer preference for wild products as a potential obstacle to substitutability. One noted: “If you can give consumers something that they want that’s a legal, sustainable alternative, then there’s no economic or psychological reason that they shouldn’t accept it. Unfortunately, we know that for a number of species, both because of the rarity effect and because of perceived differences, and in some cases because of real differences, their preference is for wild [animals]”. One interviewee reported that, in the case of turtle consumption, “there is a group of people who prefer to get turtle from the wild, because they say it’s so much tastier, and it’s healthier, and the flavor is different, and so on”. She noted that the strength of this preference for wild turtles was such that captive-bred turtles were poached from a wildlife farm and falsely labeled as wild-caught. However, another respondent who studied snakes noted that the market for wild-caught snakes “is a much smaller market [than farmed snakes] and it does not represent a threat to wild populations”.

Seven interviewees discussed how parallel markets can develop for the farmed product and the wild product. One respondent noted that, for some species, the markets for wild and farmed products are “often not even competitive, meaning that these are often parallel, separate markets; so, for these species, poaching will often increase”. Multiple interviewees contended that, in the case of bears, wildlife farms have created an additional market (for the farmed product) that does not mitigate the original demand for wild-caught animals. However, respondents also noted that the markets for farmed and wild products are often intertwined. One interviewee clarified that farmed and wild animal products “often operate together: [they are sold in the] same markets, same restaurants, [by the same] traders”. One academic said that, for certain species, a lack of labeling leads to a single market. Speaking of giant cane rats, she said, “they don’t put a label on it they just put the animal on the table…you don’t see if it was from the wild or reared in the back garden”.

#### 3.2.8. Governance

Finally, the interviewees also mentioned how governance influences the relationship between wildlife farms and demand for wildlife products. One respondent noted that good governance is “not only a prerequisite, it’s *the* prerequisite [for wildlife farms to have benefits]. In order for farming to have a conservation benefit, it goes far beyond just choosing a species that breeds like a rabbit in captivity. There has to be very strict laws and enforcement in place to prevent continual hunting or poaching of the wild populations. There has to be traceability to ensure that the retailers and consumers can ensure that the product they’re buying is from the farm and not the wild”. That interviewee also emphasized how, even when parallel markets of farmed and wild products occur, good governance can help prevent poaching. He gave the example of salmon farms which have led to “a massive increase in [farmed salmon] consumption from the United States but we haven’t, including because of good governance, we haven’t seen an increase in demand for wild salmon. Or we have seen an increase in demand for wild [salmon] but we’ve controlled it because we have a decently good regulatory system and strong enforcement”. According to another respondent, snake farms in Asia are also “quite well-regulated”.

However, other academics noted that, when strong governance is absent, wildlife farms can have counterproductive effects on wild populations. Two respondents mentioned species where captive breeding has helped wild populations in countries with strong governance but has failed to protect wild animals in Southeast Asia, where wildlife farms are prevalent, “the governments don’t always have that much power” and/or conservation regulations are not adequately enforced. For example, one academic discussed crocodile farming: “in Australia, that’s had a bit of success; in Southeast Asia, farming has not been helpful for wild crocodile populations…in fact, in Southeast Asia, I can’t think of one example that has helped wild populations”. Another interviewee gave the example of Indonesian cockatoos. These birds “are bred in captivity in the US and the US trade in cockatoos is now almost completely relying on locally bred birds so there’s no negative impact [from the US pet trade] on Indonesia’s populations anymore, but cockatoos are still poached in huge numbers in Indonesia and laundered through countries like the Solomon Islands and other countries to support demand in countries that don’t have strict legislation like the US does”.

The academics also mentioned that good governance of wildlife farms requires resources to differentiate between farmed and poached products. As one interviewee noted, when “there’s a legal source of animals that makes it really challenging to arrest someone that might have an illegal wild turtle because we can’t really tell which is which by looking at it. And I think that’s quite a normal concern to have. What they are trying to do in the Cayman Islands, they are trying to make sure that every time you buy legal turtle, it comes in a certified bag with a lock or something like that. And you have to keep it closed until you cook it or eat it”.

Although several respondents discussed the current lack of good governance in wildlife farms and the resultant laundering (which can occur when poached animals are falsely presented as legally farmed), they differed in their interpretation of laundering. Some construed laundering as primarily a governance issue; for example, one respondent said: “I can think of lots of examples of laundering. Now does the fact that there’s laundering, does that mean that wildlife farming cannot reduce wild demand? No, it means that there’s poor governance. Just like, we have taxes to collect in Indonesia and a lot of people either pay bribes or don’t get collection of taxes, they get around it, and that happens in the States, does that mean that collecting taxes is automatically bad? No, it means that there’s a governance failure around it”. In contrast, other academics viewed laundering as endemic to legal wildlife farms.

### 3.3. Stigmatization or Acceptance of Wildlife Farms

In their discussion of stigma(s), the respondents noted that stigmatization can have both detrimental and protective effects on wildlife. A few respondents mentioned the harmful effects of stigma within the conservation community. One noted that “there is a huge stigma or misperception that just because it’s [the animal’s] wild, it must be endangered”, which restrains acceptance of wildlife farms. Another academic said that, within the conservation community, sustainable use is stigmatized by some and animal welfare/rights is stigmatized by others. He noted that this can limit discussions of tools to protect wildlife, which in turn can harm conservation efforts.

However, five academics also noticed the benefits of stigma in the context of wildlife farms. One interviewee remarked on how bans can create a protective stigma: “there was a huge stigma on elephant ivory but then it started to become legal again and I think people saw it as elephants are doing just fine, and that stigma of using ivory has actually decreased and elephants are now killed in the tens of thousands again”. Other academics noted that stigma can create limits to consumption that help keep it within sustainable limits: one noted that, “when you’re speaking about wildlife consumption, I think it’s good to have some barriers about what’s OK to do and what’s not OK because otherwise it creates so much demand that you can’t sustainably meet that demand. So, in a way it’s good that [in the Cayman Islands], they still see sea turtle, even if it’s farmed, as something that is consumed occasionally, because if they wanted to consume sea turtle every day, that’s completely unsustainable”. The interviewees mentioned numerous factors that influence the stigmatization or acceptance of wildlife farms: the label “wildlife farm”, what the stigma is attached to, cultural differences in wildlife use, consumer knowledge and motivations, geopolitical factors, and demand reduction efforts (Table 5).

#### 3.3.1. Wildlife Farm Label

The respondents discussed how the label of “wildlife farm” itself can affect how a facility is perceived. One interviewee noted how the term “farm” is applied to bear bile farms in an attempt to legitimize these facilities. However, he noted that these “farms” rely solely on wild-caught animals, noting that “bear farms don’t breed bears, the bears are not going to ever breed in the cages they’re in… many of them can’t even stand up, let alone breed. Some of them are kept in metal suits so they can’t move, they can move their head and that’s it. We visited every one of them…there’s not one bear farm in Southeast Asia that breeds bears”.

Another respondent gave the example of how, for a sea turtle farm in the Cayman Islands, the phrase “wildlife farm” is purposefully omitted in order to prevent social disapproval: “Before, the name of the facility was the Cayman Sea Turtle Farm or something like that but eventually due to marketing they changed the name, so now there’s a name like Cayman Islands Water Something…it doesn’t say anything about farming…so sometimes people go there on boat cruises or something like that and they don’t really realize it’s a wildlife farming facility as well…and what they find a bit strange is that if they visit the restaurant, they serve a turtle burger and I think that probably raises some questions and then they realize ‘oh yes they are farming turtles.’ But it’s not in the name anymore. And that’s all due to the business side and knowing that if people see very quickly that it’s a wildlife farming facility, they may be a bit put off”.

One respondent mentioned how the wildlife farm label can also be greenwashed, or promoted as conservation-friendly when it’s not, he noted that “a lot of importing countries like to say that they’re importing captive-bred animals so that they don’t have a negative impact on wild conservation. So, the exporting countries pick up on that and say that all these animals that were wild last year are captive-bred this year and they basically change the wording on the paperwork and [then] the importing countries feel like they’re doing something good”.

#### 3.3.2. Source of the Stigma

The respondents also mentioned how the process of stigmatization depends upon the characteristic of wildlife consumption that the stigma is attached to. Some interviewees discussed how, for certain charismatic wildlife species, the stigmatization of wildlife farms reflects a broader social disapproval of use of that species. For example, one academic described the contrast in stigma for crocodile and turtle farms: “for example, crocodile farming, I haven’t looked at the number but there are so many farms across the world and people don’t really think twice if they’re using crocodile skin shoes. Personally, maybe I wouldn’t use them, but I think it’s more mainstream than using anything turtle related…it’s the stigma around some species. I think in people’s minds it’s OK to farm some species and not others and that makes a big contribution to why some species are on the increase and others aren’t in terms of wildlife farming…sea turtles they are a very charismatic species that people are very fond about so there’s a real emotional and cultural attachment to sea turtles so it’s not something that people support. So, it [the turtle farm] is the last one and I would not expect to see other facilities starting sea turtle farming”. Another respondent noted: “people who consider tiger farming absolutely abhorrent will enjoy wild salmon or farmed salmon without giving it a second thought”.

Other interviewees gave examples where stigma is attached to the type of production (farmed or wild-caught). For example, one respondent, speaking of snake products, explained that “in countries where animal welfare and conservation is a higher social concern, they are worried about where they get their product from. For example, one watch company in Switzerland, they are very concerned about where their leather wristbands are coming from…they do not want illegally sourced or wild-caught snakeskin wristbands on their product”. One respondent discussed how stigma can also emerge not from misgivings about the ethics of a mode of production, but out of the perceived inferiority of the wildlife product: “the reason bear bile is a failure is that consumers believe that the quality of the bear bile from the farms is not very good. And the same for rhino. And the same for tiger. They don’t care much about the farming, they just care about what they get in terms of the quality and the price”.

#### 3.3.3. Cultural Differences

The interviewees discussed how the decision about whether or not to allow wildlife farms “depends upon local attitudes and social norms”. They noted how cultural differences impact a variety of views, such as “views about what can and can’t be eaten, welfare standards, what types [of production and consumption] are OK with which types of species, and whether or not habitat and livelihood considerations should come into this”. One academic emphasized that it is important to understand these cultural differences because “wildlife farming is happening in other cultural contexts…and it’s going to happen in those other countries’ contexts because of sovereignty”. He added that the stigmatization or acceptance of wildlife farms is “not endemic to production systems” but rather a reflection of “different [cultural] relationships to animals”.

The interviewees mentioned that one area of cultural difference concerns which species are acceptable to consume and that, particularly in Southeast Asia, wildlife consumption and wildlife farms are intertwined. As one academic noted: “if an animal is being consumed in Southeast Asia, it’s 99% likely it’s being farmed” as wildlife farms are “unfortunately very integrated into the legal wildlife trade there”. Other respondents described how the consumption of numerous wildlife species are normalized. One said that: “snakes, or at least reptiles, are as much a cultural norm in Asia as chickens or pigs in more temperate climates…this is a species that’s been on the menu for millennia”. Another academic commented: “for most of the Western countries, the use of rhino horn is stigmatized, but in Asian countries like China, Hong Kong, and Vietnam, the use of animals in traditional medicine has been there for thousands of years and it doesn’t have any stigma”.

The interviewees also described how cultural norms often yield attitudes towards wildlife that are counterintuitive to someone from another culture. One academic discussed how Buddhism is a prominent religion in Southeast Asia and described how it views the correct treatment of wildlife: “in Buddhism, the correct treatment of animals is simply to let them live a life and if that life is awful, they’re still living a life…so that is correct treatment”. She also noted that, in Laos, “people were overwhelmingly, ‘oh I absolutely love bears, and I use bear bile.’ It is hard for people in a Western context to understand but it makes perfect sense for them because they have always used the resources of the forest, and you can love a tree and cut it down”.

Some respondents mentioned how, when cultural traditions are tied to wildlife consumption, wildlife farms can be viewed as a mechanism to preserve those traditions. One interviewee gave the example of turtle farming in the Cayman Islands: “the stories they [local people] told me is that initially in the Cayman Islands, it wasn’t easy to farm any other animals or fruits or anything like that and so they were using sea turtles as a very important source of protein. And so, anyone who is very connected to the history of the Cayman Islands, and connected to the sea, they remain attached to that”. As another academic noted, when wildlife species used for cultural traditions are being decimated in the wild, “you can either destroy that tradition, you can let that species go to extinction and destroy that tradition also because there’s nothing left, or you find an alternative, which sometimes is promoting use of other species, but wildlife farming kind of emerges as a possibility there”. He continued, “it’s much easier to produce than it is to change demand. And also, when demand is culturally tied, there’s perhaps very little desire to change demand. Part of what we do, who we are, what we want…It’s very easy for the United States to ban ivory or to say we don’t farm tigers, because there’s not really any tradition of doing that, but decide that we are going to ban turkeys or deer hunting…then there are a lot of people that would have problems with that”.

Respondents noted that, in countries with heterogenous cultural traditions, the process of stigmatization or acceptance of wildlife farms may look different than in more culturally homogenous contexts. One academic said: “in Asia, there are some countries that are fairly homogenous (in Japan and South Korea, people have fairly similar norms and beliefs) but other countries in Asia (such as China or Vietnam) are heterogenous and so there are different groups of people with very different social norms, values, and beliefs. For example, in China, one group might think there’s no problem, no stigma, with using tiger bone, but for other groups, such as young people, they might find a stigma. So, the stigma depends upon the society. In a homogenous society, the stigma is general for the whole population but, in a heterogenous society, a stigma might only apply for a particular group of people”. Another respondent described the diverse culture of the Cayman Islands, where the government has promoted turtle farms but where turtle consumption is highly stigmatized within certain segments of the populace: “it’s such a small island and you have so many nationalities there. You have a lot of Americans, British people, Jamaicans, so people with many different perceptions about what’s socially accepted in terms of turtle farms. But in terms of the government, of course, the government is Caymanian, and they always thought this [turtle farming] was very important for them in terms of their identity as a country. The type of issues that were discussed if you were speaking with someone local or someone from the States or UK it was so different, it’s like you were speaking almost about different topics. If it was someone local, especially someone older who was local, they would speak about the history, they would speak about the cultural value, and even show you pictures of how it was back in the day and maybe they’re even giving you recipes or telling you what restaurants you should go to get the best turtle…that’s the type of things they were telling me. If you were speaking to a British citizen there, they were saying ‘oh yes, it’s awful, the animal welfare, I would never do that’ and often they would say ‘I don’t even understand how someone would be willing to eat turtle’”.

#### 3.3.4. Consumer Typology

While respondents described how stigmatization can occur at the societal level, they also gave examples of how it can differ among segments of the population. One interviewee noted how different motivations for wildlife use affect stigma creation: “if people are doing it out of need, then stigmatize it all you want, it’s probably not going to work. And if people are doing it because they’re bad boys who don’t really care what anybody thinks, you might actually increase their desire to do it, so I think that stigma’s relationship to motivation is a little bit more complicated and has to be really targeted and tailored rather than some generic ‘this is bad’”. This respondent explained that how the consumer relates to the product (Is it necessary? Is the illegal aspect the main draw?) will impact both consumption patterns and stigmatization.

Other interviewees highlighted that, at times, the lack of stigmatization around wildlife farms is due not to attitudes or cultural beliefs but to a dearth of awareness (in both producer and consumer countries) about the practice. One interviewee noted that in consumer countries, “there’s a real ignorance” of “believing that everything is captive-bred and therefore feeling OK with buying wildlife products just because they’re declared as captive-bred. Or without understanding the consequences or the impacts of breeding that species on wild populations”. Another respondent described how, in producer countries, “the first step [to stigmatization] is to make people aware of what’s going on”, that alone “can already establish a stigma because a lot of people don’t have an opinion about these practices because they don’t know much about it”. One academic mentioned how, when “people are not aware that the practice occurs in their country or don’t care to know the extent of it”, “governments make the main decision” about the stigmatization or acceptance of wildlife farms in their countries.

#### 3.3.5. Geopolitical Factors

The interviewees reported several geopolitical factors that influence decisions about wildlife farms and described how this choice sits at the intersection of international and national forces. A common response was that wildlife trade is governed “by international agreements like CITES [the Convention on International Trade in Endangered Species of Wild Fauna and Flora]. If a country is a signatory to one of those agreements, then they need to follow that, that decision is governed or met by the local government in terms of national laws and policies”. However, some respondents emphasized how the stigmatization of wildlife consumption can differ significantly between the international and national levels, which can create conflict over wildlife farms. One academic gave the example of the last sea turtle farm in the Cayman Islands: “when the turtle farm was created, it was still legal to trade sea turtle internationally…but at the same time, some very important international decisions were made regarding the international trade of sea turtles… so there was let’s say a big fight because at that time, the Cayman Turtle Farm was claiming that their turtles were completely bred within the Cayman Islands and that they weren’t relying on wild animals anymore so they shouldn’t be seen as a wildlife farming facility…it was saying that yes, initially we took animals from the wild but now everything is bred locally… so it’s almost like they wanted to see this as domestic and it’s like selling cow—there’s no problem, this is not wildlife. It’s almost like they wanted to be seen as outside of those agreements [CITES] and [saying that] we can still do it. Once in a while there are petitions about this and once in a while, when there are CITES meetings, people still discuss this. But clearly there was a lot of government support locally within the Cayman Islands to have this as a legal activity”. This respondent described how local resistance to the enforcement of international agreements can lead to the acceptance of wildlife farms for domestic consumption even when its stigmatized at the global level.

Other interviewees outlined how political influence or enmity between countries can affect stigma. One described how, if wildlife farms are viewed as a success in one country, other countries might attempt to emulate them: “in Vietnam, [bear bile] farming has been phased out but we are worried that if people argue that bear farming is working in China, then Vietnam might go back to legalizing bear farms”. She expressed how, on the other hand, political animosity between powers can lead to a stigmatization of farmed wildlife products. In her discussion of bear bile farms, she mentioned that, “in Cambodia, there is a slight stigma because they don’t really understand farming, so to them it’s fake, it could be fake [bile]. And it’s something Vietnamese, and the Cambodians hate the Vietnamese. So, I think in that sense it’s a stigma. They’re not particularly fond of the Chinese either and now the Chinese do stuff like that [farming wildlife]…that’s probably why farmed bear bile hasn’t become a thing in Cambodia, because they’re so against the Vietnamese”. She explicated how, although this stigma emerged due to conflict between human populations rather than the treatment of wildlife, it still attached to the farmed wildlife product and affected its social acceptability. However, she also clarified that sometimes stigma affects consumption but not production: “the Vietnamese hate the Chinese and will not take Chinese bear bile in Vietnam. However, it goes the other way… one of the [Vietnamese] bear farmers I spoke with sends all his bear bile to China”.

The interviewees also explained how, since some wildlife farms function as tourist attractions, tourism is another site for the intersection of international and national influences on stigma. One respondent mentioned how a potential issue with wildlife farm tourism is that it could create more demand. In her discussion of a sea turtle farm in the Cayman Islands, she said: “because it’s such a touristic place, there were concerns about restaurants that were serving turtle meat to tourists, because they were saying that was creating more demand. Basically, we have the turtle farm that is producing some meat for domestic use, and yes the tourists are doing it there so it’s still domestic, but it’s not like they [the tourists] are doing it because of their cultural tradition. It’s just because they’re trying something new…so you’re increasing demand without really needing to increase demand”. However, this same academic mentioned how, if tourists approach wildlife farms with different notions of appropriate wildlife treatment than the local population, tourism can also increase stigma of wildlife farms. Still speaking of the sea turtle farm, she continued: “because people go and see pools with a number of animals, some of them showing stress and showing some of these bites, and I think that’s why some of these issues [objections to the farm] came to be because it’s a place where people go to visit and they see for themselves. Just by having that [tourism], that’s why people are aware of why it’s not the best for the animals”.

#### 3.3.6. Demand Reduction Campaigns

Finally, the respondents mentioned deliberate efforts to target wildlife consumption through demand reduction campaigns that attempt to alter social preferences and practices. The interviewees emphasized that much of wildlife consumption has a social component: one gave the example of how “bear bile is usually transmitted socially in terms of it’s given to you by your friend or family member to treat some form of ailment”.

The interviewees emphasized how demand reduction must have cultural resonance in order to avoid counterproductive effects. One academic discussed how stigmatization of consumers can actually prevent behavior change: “others think ‘when I’m talking to consumers, I’m talking to a criminal’ or something like that but for me, I respect everybody, my role is to provide insights so that they can make decisions, so I respect everybody: even the people who use rhino horn, I want to find a solution for them, I want to find a solution to conserve the rhino…when conservation organizations address demand reduction, when they think about traditional medicine, they always think that the product has no benefits and that the consumer is stupid…but when you talk to local people, they feel insulted by some of these campaigns. For example, the campaign that ‘rhino horn is not medicine’ has a lot of problems in Vietnam and has created objections from the local government…this doesn’t take into account cultural differences, history, social aspects…they didn’t talk to the local people or the consumers or TCM [Traditional Chinese Medicine] practitioners, and that campaign did not have effects on consumers, and in some ways created outrage from the local people”. Another respondent noted that “sometimes the message [about wildlife consumption] not only isn’t culturally appropriate to what actually changes people’s behavior, what they care about, but sometimes it’s actively counterproductive…alienating people is generally not a great way to get them to change their behavior. In any sector. It leads people to double down”. The interviewees described how, if stigmatization alienates consumers, and/or is not achieved through cultural resonance, it can increase the acceptance of wildlife consumption and farms.

However, another academic mentioned a demand reduction campaign around bear bile use in Cambodia that took these factors into account. She said: “Western medicine is very accessible in Cambodia. Our research shows that people strongly value Western medicine. We’re going the route of emphasizing Western medicine. We’re saying: ‘trust a professional doctor, don’t listen to the wrong advice.’ That’s our message. We’re not saying anything bad about traditional medicine. We also don’t mention that bears are declining because that doesn’t seem to resonate, and we don’t mention welfare because that does not seem to resonate at all”. She outlined how, while the aim of this campaign was to reduce the social acceptability and use of bear bile (both farmed and wild), it relied on culturally resonant messages and focused on promoting Western medicine rather than denigrating Traditional Chinese Medicine (TCM). Overall, the interviewees described how demand reduction campaigns intersect with many of the other factors mentioned (such as geopolitics, cultural attitudes towards animals, and consumer typology) to influence the stigmatization or acceptance of wildlife farms.

## 4. Discussion

This paper has used qualitative interview data with key informants to analyze (a) the perceived harms and benefits of wildlife farms and (b) which factors influence the stigmatization or acceptance of wildlife farms. The results indicate that respondents evaluate the harms and benefits of wildlife farms through a number of factors: animal welfare, environmental impacts (on poaching, wildlife laundering, waste, genetics, land management, and climate change), issues of scale and purpose (sustenance or commercialism), demand, accessibility and consumer preferences, species differences, substitutability, and governance. The interviewees described how these perceived harms and benefits are interpreted and acted upon within diverse cultural, economic, and political contexts, all of which influence the construction of stigma. Aspects that influence the process of stigmatization (or acceptance) of wildlife farms include the social construction of the “wildlife farm” label, what characteristic of wildlife consumption the stigma is attached to, cultural differences in wildlife use, consumer typology, geopolitical factors, and demand reduction efforts. These results have important implications for the complex and ethically fraught issue of wildlife farming.

First, the results illustrate the importance of considering legal harms when conducting an ethical analysis of wildlife farms. All of the respondents, regardless of which taxon of wildlife they studied, mentioned the detrimental consequences of wildlife farms on animal welfare. These included indications of extreme stress, food deprivation/low-quality nutrition, dehydration, limited space sometimes to the point where the animal was unable to move, inability to engage in natural behaviors (such as migration or socialization), and the separation of mothers from their offspring. These results contribute to the literature on animal welfare within the wildlife trade, which is still understudied [2]. Although most of these harms are legal within the context in which they occurred, they do violate what is known as species justice [30,31]. While these harms have significant ethical implications, they are often legal, institutionalized, and even promoted [34]. Therefore, in this case, a harm-based approach offered a broader, more comprehensive analysis of impacts than a strict legalistic vision.

This paper has also mapped which contextual factors influence the perceived harms and benefits (and resultant ethical implications) of wildlife farms. The interviewees gave examples of how even the label “wildlife farm” is subject to various social meanings. For example, respondents who studied bear bile farms noted that these facilities use wild-caught animals because the conditions of use (space restrictions and poor welfare) prevent breeding. In the case of bear bile farms, the label “wildlife farm” is inappropriate but still widely used and can be seen as an attempt to legitimize these facilities. In contrast, other wildlife farms (such as a sea turtle farm in the Cayman Islands) avoided the label “wildlife farm” to prevent alienating tourists. This contrast illustrates how the term “wildlife farm” can either serve a legitimizing or a stigmatizing function, depending upon the cultural and species context.

The purpose of wildlife farms (sustenance or commercialism) also emerged as a prominent factor that influenced how respondents evaluated wildlife farms. Overall, academics who researched sustenance-based wildlife farms, or small-scale farms that produced wildlife for food, tended to focus on the benefits of farming. In contrast, respondents who framed wildlife farms as commercial enterprises often emphasized harms. Interviewees who analyzed commercial wildlife farms mentioned how these venues often market new/expanded uses for the wildlife product (which can increase demand) and how the profit motive of these enterprises often has detrimental impacts, particularly on animal welfare. In addition, all of the respondents emphasized how species differences influence the repercussions of wildlife farms. Respondents mentioned how wildlife farms can produce differential results due to biological characteristics of the species, such as time to maturity, ability to breed in captivity, and resilience to harvest. Interviewee narratives also revealed how demand for wildlife products (and thus the effect of wildlife farms on demand) is complex and informed by numerous variables, which include consumer preferences and attitudes, species differences, substitutability, and accessibility.

Finally, this paper demonstrates how a consideration of the ethical dimensions of wildlife farms must take into account the contextual nature of stigma. The interviewees discussed how stigma can attach to multiple factors: the species, the production method, the culture/country that is associated with the product or the production method, or the perceived inferiority of the product. Stigma is also influenced by segmentation of the human population and by geopolitical factors. In heterogenous countries with diverse cultural traditions, the process of stigmatization or acceptance of wildlife farms may look different than in more homogenous countries. Views of the practices of other nations/cultures can serve to either promote or dampen acceptance of farmed wildlife products, and local resistance to international agreements can lead to the acceptance of wildlife farms for domestic consumption even when consumption is stigmatized at the global level. Tourism is a factor that further complicates stigma, as it involves cross-cultural views on animals and access to wildlife farms.

The data provided in this paper can inform policymaking related to wildlife farms through applied ethical processes such as argument analysis [41]. The steps of argument analysis are to (a) express a reason for behaving in a particular way, (b) express that reason as a formal argument, and (c) evaluate the argument through making judgments about the appropriateness of stated premises, which include both ethical and empirical premises [41]. One strength of this approach is that it can incorporate pluralistic ethical arguments (regarding animal welfare, conservation, etc.) that are made from diverse orientations (such as utilitarianism, deontology, etc.). It has been demonstrated how argument analysis can lead to ethically informed decision-making on wildlife farms in the specific case of lions farmed for their bones [41]. This paper builds on this prior work through its identification of how the arguments for and against wildlife farms (or the perceived harms and benefits) differ across contextual variables such as country, species, and scale. Policymakers can use this information to catalogue and evaluate (through argument analysis or a similar process) the different assertions that inform the wildlife farm debate, with the added knowledge of how these contentions vary across contexts.

## 5. Conclusions

Despite the contributions of this paper, there are several limitations of the research that could be addressed in future studies. The exclusion of the terms “game ranching” and “wildlife ranching” from the sampling criteria means that the geographic focus leaned more towards Asia than towards North American or South African contexts. It would thus be helpful to replicate this work with a focus on “ranching” facilities to see what potential differences emerge. Further, the interview protocol mentioned “conservation” early in the interview process which may have led respondents to overly focus on conservation impacts as opposed to other factors. Finally, the small sample size limits generalizability but does provide detailed qualitative data that can be expanded upon in future research.

This work has discussed how key informants conceptualize the harms and benefits of wildlife farms in order to provide a roadmap of which factors to consider in decisions on this contentious practice. Further, it has discussed the process by which wildlife farms are stigmatized or accepted. Stigmatization impacts what uses of wildlife are labeled detrimental (harms/crimes) and which are deemed allowable. Information on stigmatization illuminates how shifts in context alter the perceived ethical repercussions of wildlife farms. These shifts include variability in wildlife species, notions of the proper treatment of animals, geopolitical factors, and even the meaning of “wildlife farm” itself. This paper has offered a comprehensive qualitative analysis of the cultural, economic, and environmental complexities of wildlife farms such that researchers and other stakeholders can understand the impacts of this practice on species, communities, individual animals, and the wildlife trade, both legal and illegal.

## Figures and Tables

**Table 1:** Sample.

Respondent	Wildlife Species of Expertise	Geographic Focus
1	Snake	Asia
2	Rhinoceros	Asia; Africa
3	Bear, Tiger	Asia
4	Numerous Fauna	Numerous Continents
5	Giant Cane Rat	Africa
6	Sea Turtle	Oceania
7	Numerous Flora and Fauna	Asia
8	Bear, Pangolin, Numerous Fauna	Asia

**Table 2:** Respondents’ Definitions of Conservation.

“The preservation of ecological integrity both in its optimal form and in varying compromises associated with anthropogenic integrity”.
“Preservation of natural resources”.
“Active management that aims to preserve biodiversity and ecosystem functioning”.
“Maintaining the environment or maintain any natural assets such that they are also available to future generations”.
“Maintenance or improving of the conservation status of the species”.
“Efforts to protect ecosystems and species but across a wide range of strategies…including highly protectionist through to sustainable use”.
“Successful efforts to keep sustainable populations in a functioning role in their native habitats”.

**Table 3:** Respondents’ Definitions of the Correct Treatment of Wildlife.

**Cultural Factors**
“Strictly science-based but with some qualifications…different cultural perspectives have to be taken into consideration”.
“For me, conserving the rhino and protecting the rhino is equally as important as the health of the local people”.
“In Buddhism, the correct treatment of animals is simply to let them live a life and if that life is awful, they’re still living a life… so that is correct treatment”.
**Animal Welfare Factors**
“[We should] consider that animals also feel stress and pain; unfortunately, our religion and culture have often taught us differently… [I] look at it from a moral point of view”.
“Ideally I would like for individuals [individual turtles] not to suffer”.
“Fair and ethical treatment of individuals [individual animals]”.
**Economic Factors**
“I’m trained to look at animals as the path of productive capital that you maintain and harvest in order to generate income”.
“I would consider the value of the industry…is it an industry that’s employing a lot of people…is it a useful or a beneficial industry?”
**Conservation Factors**
“My first consideration would be the impacts on wild populations. And impacts on other species, such as prey and predator”.
“What is good for the common good of the species… I don’t agree personally that wildlife farming is correct morally but if I can see the evidence and the project is leading to an improved state for those animals in the wild then I’m willing to make that trade off”.
**Criminal Factors**
“I would consider the criminal element…involved in wildlife farming in some places there’s a criminal element, an organized crime element”.

**Table 4:** Factors Used to Evaluate the Harms and Benefits of Wildlife Farms.

**Animal Welfare**
**Environmental Impacts**
Poaching
Laundering
Waste
Genetics
Land Management/Climate Change
**Sustenance/Commercialism/Scale**
**Demand**
**Accessibility/Consumer Preferences**
**Species Differences**
**Substitutability**
**Governance**

Bold font indicates a category of response and un-bolded font delineates a sub-category.

**Table 5:** Factors that Influence the Stigmatization or Acceptance of Wildlife Farms.

**The “wildlife farm” label**
**What is the stigma attached to?**
Species
Type of production
Perceived quality of product
**Cultural differences in wildlife use**
Attitudes/norms
Traditions
Cultural heterogeneity or homogeneity
**Consumer typology**
**Geopolitical Factors**
International or domestic consumption
Politics
Tourism
**Demand reduction efforts**

Bold font indicates a category of response and un-bolded font delineates a sub-category.

## Appendix A: Interview Questions

**Question 1:**

How would you define *wildlife farming*? Which species and what geographic areas are your areas of expertise? Is wildlife farming expanding, decreasing, or remaining constant?

**Question 2:**

How do you evaluate the pros and cons of wildlife farming?

**Question 3:**

How do you define conservation?

**Question 4:**

How do you define the “correct” treatment of wildlife? What do you use to make this decision? Economic considerations? Moral, religious, cultural, or philosophical factors?

**Question 5:**

Does wildlife farming *harm* wildlife? If so, how would you describe that harm?

**Question 6:**

In your work, do you gather data on the well-being or suffering of individual animals? If so, please describe if wildlife farming leads to any of the following:

- Disease, injury or functional impairment?
- Environmental challenges such as exposure to extreme temperatures or injurious housing conditions?
- Behavioral or interactive restrictions, such as the inability to exercise natural behaviors or interact with other animals?
- Anxiety, fear, pain, or distress?
- Food deprivation, water deprivation, or malnutrition?

**Question 7:**

Can you describe the methods used to farm wildlife? Does the profit motive (the need to maximize profit) affect the techniques used to farm wildlife? If so, how?

**Question 8:**

What economic, social, cultural, or environmental factors have contributed to the rise of wildlife farming, to the perpetuation of wildlife farming, and to its legal status?

**Question 9:**

Who decides if wildlife farming is legal or illegal? How is this decision made? What factors are considered when making this decision? Economic factors? Cultural factors? Animal rights or welfare? The conservation status of the species?

**Question 10:**

What do you see as the connection between poaching and wildlife farming? In your opinion, does wildlife farming increase, decrease, or have no impact on poaching? What, if any, do you see as the difference between an animal killed legally on a wildlife farm and an animal poached from the wild?

**Question 11:**

Does your work involve interaction between people from different cultures and/or countries? If so:

- How would you describe this interaction?
- Have you encountered cultural differences (either between your colleagues or between your colleagues and members of the local community) in notions of conservation? In notions of animal rights? In attitudes towards wildlife farming? In attitudes towards wildlife consumption? If so, how do you navigate cultural differences?
- How do you set research or advocacy agendas?
- How do you decide the organization’s position on wildlife farming?

**Question 12:**

Is there a stigma surrounding wildlife farming? If so, is the presence or absence of stigma a benefit or a detriment to the species? Is the presence or absence of stigma a benefit or a detriment to individual animals? Would you like to see the level of stigma surrounding wildlife farming increase, decrease, or remain the same? What do you think would need to change to increase, decrease, or maintain the current level of stigma surrounding wildlife farming?

**Question 13:**

In your opinion, how does wildlife farming affect the demand for wildlife products? Does wildlife farming increase demand, decrease demand, or have no effect?
